# RNA-Seq transcriptomic profiling of primary murine microglia treated with LPS or LPS + IFNγ

**DOI:** 10.1038/s41598-018-34412-9

**Published:** 2018-10-31

**Authors:** Marta Pulido-Salgado, Jose M. Vidal-Taboada, Gerardo Garcia-Diaz Barriga, Carme Solà, Josep Saura

**Affiliations:** 1 Department of Biomedical Sciences, Biochemistry and Molecular Biology Unit, School of Medicine, University of Barcelona, IDIBAPS, Barcelona, Spain; 2 Department of Biomedical Sciences, Histology Unit, School of Medicine, University of Barcelona, IDIBAPS, Barcelona, Spain; 3 Department of Cerebral Ischemia and Neurodegeneration, Institut d’Investigacions Biomèdiques de Barcelona, CSIC, IDIBAPS, Barcelona, Spain; 4 Institute of Neurosciences, University of Barcelona, Barcelona, Spain; 5 Peripheral Nervous System, Neuroscience Dept, VHIR- Vall d’Hebron Research Institute, Barcelona, Spain

## Abstract

Microglia, the main resident immune cells in the CNS, are thought to participate in the pathogenesis of various neurological disorders. LPS and LPS + IFNγ are stimuli that are widely used to activate microglia. However, the transcriptomic profiles of microglia treated with LPS and LPS + IFNγ have not been properly compared. Here, we treated murine primary microglial cultures with LPS or LPS + IFNγ for 6 hours and then performed RNA-Sequencing. Gene expression patterns induced by the treatments were obtained by WGCNA and 11 different expression profiles were found, showing differential responses to LPS and LPS + IFNγ in many genes. Interestingly, a subset of genes involved in Parkinson’s, Alzheimer’s and Huntington’s disease were downregulated by both treatments. By DESeq analysis we found differentially upregulated and downregulated genes that confirmed LPS and LPS + IFNγ as inducers of microglial pro-inflammatory responses, but also highlighted their involvement in specific cell functions. In response to LPS, microglia tended to be more proliferative, pro-inflammatory and phagocytic; whereas LPS + IFNγ inhibited genes were involved in pain, cell division and, unexpectedly, production of some inflammatory mediators. In summary, this study provides a detailed description of the transcriptome of LPS- and LPS + IFNγ treated primary microglial cultures. It may be useful to determine whether these *in vitro* phenotypes resemble microglia in *in vivo* pathological conditions.

## Introduction

Microglia are the main cells of myeloid origin in the central nervous system (CNS). They are considered tissue-resident macrophages that constantly patrol the cerebral microenvironment and respond to pathogens and cell damage^1^. Microglia responses to changes in homeostasis are highly plastic and multifaceted: finely tuned by the nature of the disturbance^2^. One of the most widely used stimuli to elicit inflammatory microglia is lipopolysaccharide (LPS) alone or in combination with interferon gamma (IFNγ).

LPS is the main structural component of the outer membrane of Gram-negative bacteria. It is a heat-stable, amphiphilic molecule comprising three regions: lipid A, a core oligosaccharide and an O side chain^3^. The primary receptor of LPS is Tlr4, which is a pivotal contributor to the microglial response to endotoxins, and is involved in LPS-mediated neurodegeneration^4^. *In vitro*, LPS-treated microglia produce pro-inflammatory mediators, such as Tnf, Il-1β, prostaglandin E2, NO and reactive oxygen species (ROS) (reviewed in Schwartz *et al*.^5^). LPS has been used to study the involvement of neuroinflammation in experimental models of Parkinson’s and Alzheimer’s disease, as well as amyotrophic lateral sclerosis (reviewed in Ransohoff & Perry^6^ and Nayak *et al*.^7^). Specifically, neuron-microglia co-cultures treated with LPS are a powerful tool to study the mechanisms involved in the dopaminergic neuronal death observed in Parkinson’s disease. *In vivo*, injection of LPS into the substantia nigra also results in the progressive and irreversible loss of dopaminergic neurons. In both cases, LPS triggers rapid activation of microglia, which precedes neuronal death. Therefore, LPS-treated microglia constitute a suitable system for the screening of therapeutic targets against neurodegeneration^8^.

IFNγ mediates immune and inflammatory responses by activating transcription factor STAT1 through the JAK-STAT signalling pathway. Interestingly, many IFNγ functions are mediated by cross-regulation of cellular responses to other inflammatory stimuli^9^. Therefore, IFNγ has been used together with LPS to mimic a microglial activation state that could be induced *in vivo* by the co-presence of damage-associated molecular patterns acting on TLRs, together with IFNγ produced by CNS cells. LPS + IFNγ treatment induces the production of pro-inflammatory mediators in the BV2 microglial cell line and in primary microglial cells^10^. However, whereas in some cases IFNγ potentiates the effect of LPS, e.g., NO production; in others, IFNγ counteracts it, e.g., Ptges and Csf3 expression^11^. These results indicate that two stimuli are capable of activating different pathways and they are not redundant.

Since modelling neuroinflammation and microglial activation *in vitro* constitutes a valuable tool to study the role of microglia under normal and pathological conditions, it is important to know how LPS and LPS + IFNγ affect microglia. In order to analyse the expression pattern of genes affected by these stimuli, we performed RNA-Sequencing analysis (RNA-Seq) on primary microglial cells stimulated with LPS or LPS + IFNγ. RNA-Seq is increasingly used to determine gene expression, since it provides unbiased expression profiles, identifies novel transcribed regions and is extremely accurate in comparison to RNA microarrays^12^.

Different methodologies have been developed to analyze RNA-Seq data in recent years. Here, we use DESeq to detect differentially expressed genes (DEGs), i.e., genes that are significantly upegulated or downregulated in pairwise comparisons, in murine primary microglia treated with vehicle, LPS or LPS + IFNγ. Additionally, we also use a different data analysis approach termed weighted gene correlation network analysis (WGCNA), which measures the correlation among genes, regardless of significance. Furthermore, since this approach compares all the genes in the data, it can infer complex expression patterns that could easily be overlooked in the pairwise differential expression analysis. Both, DESeq and WGCNA revealed that not all genes are equally affected by LPS and LPS + IFNγ. Therefore, our results confirm that the two stimuli generate distinct gene expression patterns and can constitute useful tools to induce different profiles of inflammation in microglia.

## Materials and Methods

### Animals

Phenotypically wild-type C/EBPβ^fl/fl^ mice on a C57BL/6J background (Jackson Laboratories) were breed and housed under specific pathogen-free conditions in the animal facilities at the School of Medicine, University of Barcelona. They received food and water *ad libitum*. All procedures were approved by the Ethical Committee for Animal Experimentation of the University of Barcelona and by the Commission for Animal Experimentation of the Generalitat de Catalunya, with protocols numbers DAAM 5026, 7064 and 7065.

### Primary microglial cultures

Microglial primary cultures were prepared as described previously^11^. Briefly, cortices from P1-P3 mice were dissected, carefully stripped of their meninges and digested with 0.25% trypsin for 30 min at 37 °C. Trypsinization was stopped by adding an equal volume of culture medium (Dulbecco’s modified Eagle medium-F-12 nutrient mixture, fetal bovine serum 10%, penicillin 100 U/mL, streptomycin 100 μg/mL and amphotericin B 0.5 μg/mL) with 160 µg/mL deoxyribonuclease I, all from Invitrogen. Cells were brought to a single cell suspension by repeated pipetting followed by passage through a 100 μm-pore mesh and pelleted (7 min, 200 g). Glial cells were resuspended in culture medium and seeded at a density of 3.5 × 10^5^ cells/mL. Cultures were maintained at 37 °C in humidified 5% CO_2_–95% air. Medium was replaced once a week. At DIV 20, mixed glial cultures were treated with diluted trypsin^13^ to obtain >98% pure microglial cultures. Microglial cells were used 24 h after isolation.

### Cell treatment and total RNA isolation

Three independent microglial cultures were treated for 6 h with vehicle (cell culture medium) and 100 ng/mL of LPS (026:B6 *Escherichia coli* serotype, Sigma Aldrich) with or without 1 ng/mL of recombinant mouse IFNγ (I4777, Sigma Aldrich). Then, total RNA was isolated by lysing pelleted microglial cells from one 75 cm^2^ flask per treatment condition with 1 mL of TriReagent (Sigma Aldrich) and 100 µl of 1-bromo-3-chloropropane (BCP, Sigma Aldrich). The aqueous phase containing total RNA was recovered after centrifuging for 15 min at 12.000 g at 4 °C, mixed with and equal volume of ice-cold 70% Ethanol and loaded onto a PureLink^TM^ Micro Kit column (Invitrogen). Total RNA was then purified following manufacturer’s instructions. Total RNA was quantified spectrophotometrically using a Nano Drop ND-1000 (Thermo Scientific) and its integrity and quality were assessed with the Bioanalyzer 2100 system (Agilent). Only samples with an RNA integrity number (RIN) greater than 8 were used subsequently for RNAseq and qRT-PCR analyses.

### qRT-PCR

300 ng of total RNA were used to carry out reverse transcription reactions with random primers using Transcriptor Reverse Transcriptase (Roche). Then, cDNA was diluted 1/30 and 3 µl were used to perform quantitative real-time PCR (qRT-PCR) with qPCRBIO Sygreen Mix Lo-ROX (PCB-P20.11–50, Vitro) in 15 µl of final volume reaction using CFX96 Thermal Cycler equipment (Bio-Rad). Measurements were performed in duplicates. Primers, shown in Table 1, were used at final concentration of 300 nM. Actin-β and Rn18s expression was not altered by treatments (data not shown). Samples were run for 45 cycles (95 °C for 30 seconds, 60 °C or 62 °C for 1 minute and 72 °C for 30 seconds). Amplification specificity was confirmed by the analysis of melting curves and by agarose-gel electrophoresis. Relative gene expression values were calculated using Bio-Rad CFX Managing software (Bio-Rad) with the comparative Ct or ΔΔCt method.

**Table 1:** Primers used in this work.

Gene	Forward (5′ → 3′)	Reverse (5′→ 3′)
Cmklr1	CAA CAG GAG CAG GGG ACC GA	TTC CTC ACC CAC GAA GAC TGC
Csf3	AGA GCT GCA GCC CAG ATC ACC	AGC TGC AGG GCC ATT AGC TTC A
Cybb	ACT CCT TGG GTC AGC ACT GGC	GCA ACA CGC ACT GGA ACC CCT
Il-6	CCA GTT TGG TAG CAT CCA TC	CCG CAG AGG AGA CTT CAC AG
Nos2	GGC AGC CTG TGA GAC CTT TG	GCA TTG GAA GTG AAG CGT TTC
Ptgs1	GTG CTG GGG CAG TGC TGG AG	TGG GGC CTG AGT AGC CCG TG
Ptgs2	TGC AGA ATT GAA AGC CCT CT	CCC CAA AGA TAG CAT CTG GA
Tnf	TGA TCC GCG ACG TGG AA	ACC GCC TGG AGT TCT GGA A
Rn18s	GTA ACC CGT TGA ACC CCA TT	CCA TCC AAT CGG TAG TAG CG
β-actin	CAA CGA GCG GTT CCG ATG	GCC ACA GGA TTC CAT ACC CA

### cDNA library preparation and RNA-Seq

Following Illumina’s (San Diego, CA) protocols, cDNA library preparation and ultrasequencing were performed. The first step was removing transfer RNA (tRNA) and ribosomal RNA (rRNA) from 1 µg of total RNA using TruSeq Stranded Total RNA Sample Prep Kits (Illumina). Then, the RNA pool (mRNA, miRNA, lncRNA and other RNAs) was fragmented into pieces of approximately 200 bp. The cleaved RNA fragments were reverse transcribed into first strand cDNA using reverse transcriptase and random primers. Next, the second strand was synthesized using DNA polymerase I and RNAseH. These double-stranded cDNA fragments were end-repaired by T4 DNA polymerase and Klenow DNA polymerase, and phosphorylated by T4 polynucleotide kinase. The cDNA products were incubated with Klenow DNA polymerase to generate Adenine overhangs, therefore allowing ligation to Illumina indexing adapters to the double stranded cDNA ends. The adapter-ligated products were purified with Ampure XP magnetic beads (Agencourt Bioscience Corpo-ration, Beverly, MA, USA) and libraries were amplified by 15 cycles of PCR with Phusion DNA polymerase (Thermofisher, Finnzymes Reagents, Vantaa, Finland). Constructed libraries were validated and quantified using BioRad’s automated electrophoresis system Experion and qPCR respectively. Pools of 6 indexed libraries were mixed (multiplexed) at equimolar ratios to yield a total oligonucleotide mix concentration of 10 nM. Finally the resulting libraries were sequenced on the Genome Analyzer IIx platform (Illumina) to generate 150 bp single reads. Six pooled indexed libraries were sequenced in each flow cell lane. Raw sequence (FASTQ format) were processed through a series of sequential steps: (1) aggressive adapters removal; (2) alignment/mapping of RNA sequences to the mouse genome reference (Mus_musculus.mm10) using *tophat* software; and (3) sorting and cataloguing of the results by using *Samtools* software^14^ in BAM files.

### Gene expression analysis using WGCNA

BAM files were processed using the Rsubread package^15^ in the R environment^16^ and summarized to the mm10 version of mouse genome with the function *featureCounts*. Summarized readings by gene were then normalized using *voom* normalization^17^ to fit the count matrix into linear modelling with the package *limma*^18^ and to obtain log2 RPKM values. Whole read counts normalized were used to cluster samples with package *hcluster* using standard hierarchical cluster with average linkage. Heatmap visualization and clustering of DEG’s was done with Genesis software after expressing gene values in standard deviation and using hierarchical clustering with average linkage by genes and samples. Genecodis^19^ tools (http://genecodis.cnb.csic.es/) were used to obtain Gene Ontology and KEGG pathway terms enrichment using the adjusted p-value < 0.01 after an hypergeometric test. Finally, Reads per kilobase per million mapped reads (RPKM) filtered values (sum of RPKM > 2 for each gene) were introduced to the Weighted Correlation Gene Network Analysis (WGCNA) package in R, to perform WGCNA as previously described^20^. Briefly, combined samples were measured with the pickSoftThreshold function to obtain a correct power β considering the smallest value to give a free-scale topology. Using 18 as the power value, the blockwise function was used with parameters mergeCutHeight = 0.2 to obtain modules containing genes with high co-expression similarity. WGCNA software then extracted the first principal component of the expression values of each module to obtain the module eigengene.

### Differential Expressed Genes Analysis

To identify DEGs, the BAM files were analyzed using R-software packages easyRNAseq^21^ and DESeq^22^. The “Coverage vectors” for all genes of each sample were obtained using “easyRNAseq” and the UCSC.mm10 Mus musculus reference genome. Samples were grouped by phenotype (WT, WT-L, WT-L + I) and the expression for each locus. To find DEGs, this count matrix was analyzed using “DESeq” software, a method based on the negative binomial distribution, with variance and mean linked by local regression. For each phenotype comparison, the group base expression, the fold Regulation, the uncorrected p-value, and q-value (false discovery rate corrected p-value) for each gene were calculated. For each comparison, the list of DEGs with a q-value < 0.05 was analyzed using Genecodis tools to obtain Gene Ontology and KEGG pathway^23–25^ enrichment as previously described. For the Signalling Pathway Impact Analysis (SPIA) of the DEGs, the Clusterview web software^26^ (https://pathview.uncc.edu/analysis) was used for data integration and visualization of alterations in several KEGG inflammatory pathways (Toll-like receptor signalling, NF-kappa B signalling, TNF signalling, Cytokine-cytokine receptor interactions) using default parameters.

### Statistical and Power analyses

Statistical analyses of qRT-PCR data were performed using one-way ANOVA followed by Bonferroni post-test. Values of p < 0.05 were considered statistically significant. Results were represented as mean ± standard error of the mean (SEM). Experimental data were analyzed using GraphPad Prism 5.01 software.

Differential gene expression and WGCNA analyses were calculated using DEseq and WGCNA statistical R-packages software. For Statistical power calculation, the RNAseqPS software^27^ (https://cqs.mc.vanderbilt.edu/shiny/RnaSeqSampleSize/) was used. Assumptions made were that prior data indicated that the minimum average read counts among the prognostic genes in the control group was 5, the maximum dispersion was 0.5 and the ratio of the geometric mean of normalization factors was 1. It was also supposed that the total number of genes for testing was 14500 and the top 300 genes were prognostic. Therefore, the present RNA sequencing experiment with 3 experimental subjects in each group can identify differential gene expression between two groups if the desired minimum fold change was 23.5. Then, it will be possible to reject the null hypothesis by using exact test with a statistical power of 80%. The FDR associated with this test was 0.01.

### Literature Search

On 26^th^ May 2018, the query terms “microglia AND RNA sequencing”, “microglia AND transcriptome” and “microglia AND RNA seq” retrieved 278, 205 and 54 results, respectively, from the Pubmed database (https://www.ncbi.nlm.nih.gov/pubmed/). The search was limited to studies published in English. A total of 7 reports have been selected according to their similarity in terms of treatment paradigm and microglial source to our study.

## Results

### RNA-Seq transcriptional analysis of primary microglia treated with LPS or LPS + FNγ

**Figure 1:**
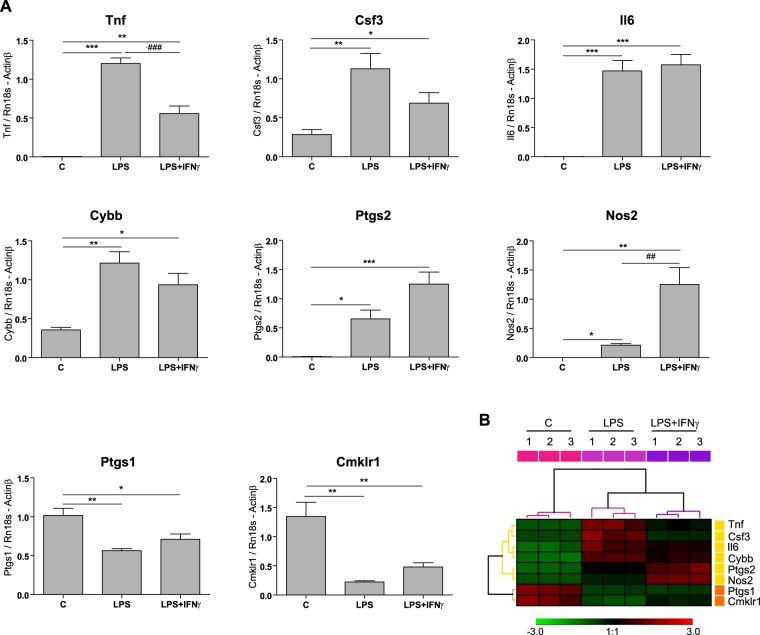
Microglial gene expression is affected by LPS alone or together with IFNγ, 6 h after treatment. (**A**) Expression of *Tnf, Csf3, Il6, Cybb, Ptgs2, Nos2, Ptgs1* and *Cmklr1* was analysed by qRT-PCR in primary microglial cultures treated with vehicle (C), LPS (100 ng/mL) or LPS + IFNγ (1 ng/mL) for 6 h (n = 4 independent experiments). Data are shown as mean + SEM. *p < 0.05; **p < 0.01; ***p < 0.001 compared with the respective control condition. ^##^p < 0.01; ^###^p < 0.001 compared with the corresponding LPS condition. (**B**) Heatmap of RNA-Seq data showing *Tnf, Csf3, Il6, Cybb, Ptgs2, Nos2, Ptgs1* and *Cmklr1* expression profiles. Values are represented as SD from the normalized average for each gene. Hierarchical Clustering (average linkage, Euclidean distance) grouped the samples into three clusters corresponding to vehicle (pink), LPS (magenta) and LPS + IFNγ (purple) conditions. Furthermore, the genes were grouped into two different clusters depending on whether the treatment increases (yellow) or decreases (orange) their expression.

It is known that LPS alone or in combination with IFNγ, two widely used pro-inflammatory treatments, triggers significant changes in the microglia transcriptome. As depicted in Fig. 1A, *Tnf, Csf3, Il-6, Cybb, Ptgs2* and *Nos2* expression is induced in microglia treated with LPS or LPS + IFNγ for 6 h, in comparison with vehicle-treated cells. However, other genes, such as *Ptgs1* and *Cmklr1* are downregulated. Interestingly, the addition of IFNγ to LPS seems to compensate the expression of some LPS-induced genes, such as *Tnf*, in a statistically significant manner; and of others, like *Csf3, Cybb, Ptgs1* and *Cmklr1*, merely as a non-significant trend. This suggests that the two stimuli are not always additive; indeed, they can be compensatory. To establish a high-resolution transcriptome profile of primary murine microglial cells in response to LPS and LPS + IFNγ, we performed RNA-Seq analysis. Three independent microglial cultures were treated with a vehicle, LPS or LPS + IFNγ; total RNA was isolated 6 h after treatment and analysed by RNA-Seq. RNA libraries and sequencing were prepared as described in the “Materials and Methods” section. Normalized data represented on a heatmap for the abovementioned genes show the same expression pattern as that observed in qRT-PCR experiments (Fig. 1B).

**Figure 2:**
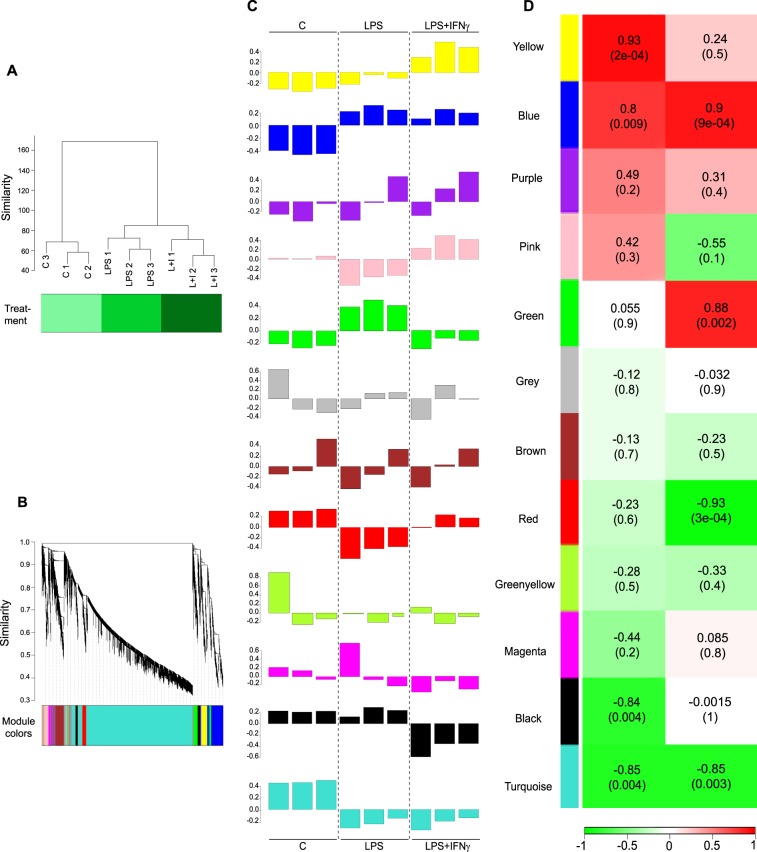
Treatment with LPS alone or together with IFNγ for 6 h induces 11 different expression patterns in primary microglia. (**A**) Hierarchical clustering of total RNA-Seq RPKM normalized values clearly separates the 9 samples according to treatment condition: Control, LPS and LPS + IFNγ. (**B**) WGCNA correlation dendrogram of genes. According to their expression pattern, the genes were grouped into 11 different modules identified by arbitrary colours, turquoise module was the largest and the green-yellow the smallest. Outliers or unclustered transcripts are grey. Genes grouped in the yellow, blue and green modules and half of the genes clustered in the black module are clustered separately from the genes present in the other modules. (**C**) Module eigengene barplots represent how expression of the genes clustered in the 12 modules was affected by treatments. (**D**) To account for the different patterns of gene expression, two models of treatment order were fitted to the WGCNA. Left column. First model of treatment order (C, LPS, LPS + IFNγ). Right column. Second model of treatment order (C, LPS + IFNγ, LPS). Correlation between gene expression and treatment effect is represented using a red-green gradient: dark-red, 1, being the most positive correlation and dark-green, −1, the most negative correlation. The correlation p-value for each module is shown in parentheses.

The strong effect of the treatments on microglial RNA expression was revealed by unsupervised cluster analysis using normalized counts (reads per kilobase million, RPKM). The 9 samples were grouped into three clearly differentiated clusters corresponding to the microglial cultures treated with vehicle (n = 3), LPS (n = 3) and LPS + IFNγ (n = 3). No outliers were detected (Fig. 2A).

### WGCNA reveals that LPS and LPS + IFNγ induce 11 different expression patterns in microglial cells

WGCNA is a method used in systems biology to find clusters or modules of highly interconnected genes and their correlation to a trait, e.g., treatment^20^. In our RNA-Seq study, we used WGCNA to analyze the gene expression patterns, in response to LPS and LPS + IFNγ, of all 14337 genes detected in cultured microglia. Our analysis clustered the genes into 11 distinct modules, corresponding to 11 different expression profiles, which were coded by arbitrarily assigned colours (Fig. 2B). The numbers of genes in the modules were, in descending order: 9343 (turquoise), 1169 (blue), 886 (brown), 532 (yellow), 516 (green), 441 (red), 432 (black), 334 (pink), 212 (magenta), 192 (purple) and 79 (green-yellow). Grey (201 genes) was reserved for outliers or unclustered genes that do not fit in any module. Branches in the hierarchical clustered dendrogram correspond to individual genes and the y-axis shows the distance between them, i.e. similarity, the more distant the less similar. Note that all genes in “blue”, “yellow” and “green” modules, as well as half of genes grouped in “black”, are clustered separately from the genes in the other modules. Furthermore, although some modules are well-defined, such as “yellow”, and most of “blue”, “brown” and “turquoise”, others are not.

The module eigengene can be used to summarize and represent the gene expression profile of a given module. Thus, barplots of the values of the module eigengene were used to represent the correlation between genes clustered within a module and the effect that LPS and LPS + IFNγ had on their expression (Fig. 2C). To account for the different patterns of gene expression, we fitted two models of treatment orders into the WGCNA. For the first one, we considered the pattern of genes that are modified by LPS, but even more by LPS + IFNγ (i.e., treatment order: C, LPS, LPS + IFNγ). This model showed the “yellow”, “blue”, “black” and “turquoise” modules to be the most strongly correlated (Fig. 2D, first column). Next, to obtain the gene patterns that were more strongly affected by LPS than by LPS + IFNγ, we fitted the reverse treatment order (C, LPS + IFNγ, LPS) and checked for the module correlation. In this case, the modules “blue” and “turquoise”, whose genes are upregulated or downregulated by both treatments, were again strongly correlated; but so were the “green” and “red” modules (Fig. 2D, second column). Therefore, genes grouped in the “yellow”, “blue”, “green”, “red”, “black” and “turquoise” modules were further analysed, to understand the biological significance of the gene expression patterns induced by LPS and LPS + IFNγ.

### Ontological analysis of WGCNA differentially affected modules

**Figure 3:**
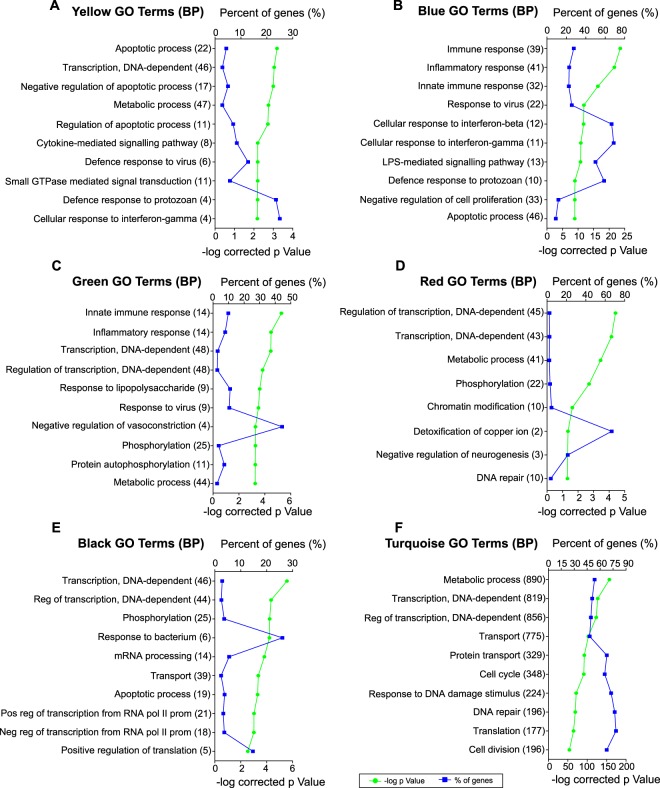
Enrichment analysis of Gene Ontology (GO) terms (biological processes) in selected WGCNA modules. This figure shows the 10 most significantly enriched GO terms in the yellow (**A**), blue (**B**), green (**C**), red (**D**), black (**E**) and turquoise (**F**) modules. Gene percentage annotation (i.e., the percentage of the total number of genes annotated in each category that were detected in this work) is depicted on the upper x-axis; whereas the p-value is represented on the lower x-axis. The biological processes and the number of genes annotated within each of them are presented on the y-axis.

**Figure 4:**
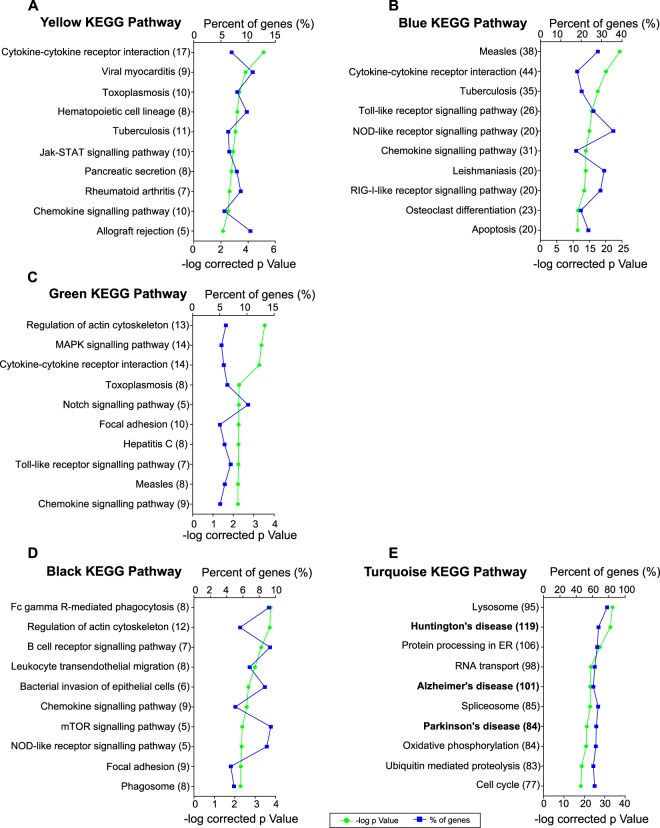
Enrichment analysis of Kyoto Encyclopedia of Genes and Genomes (KEGG) pathways in selected WGCNA modules. This figure shows the 10 most significantly enriched KEGG pathways^23–25^ in the yellow (**A**), blue (**B**), green (**C**), red (**D**), black (**E**) and turquoise (**F**) modules. Gene percentage annotation (i.e., the percentage of the total number of genes annotated in each category that were detected in this work) is depicted on the upper x-axis; whereas the p-value is represented on the lower x-axis. The biological processes and the number of genes annotated within each of them are presented on the y-axis.

The Genecodis platform^19^ was used to perform the ontological analysis of those genes clustered in the “yellow”, “blue”, “green”, “red”, “black” and “turquoise” modules. Specifically, we focused on Gene Ontology (GO) terms for Biological Process (BP) (Fig. 3) and on Kyoto Encyclopedia of Genes and Genomes (KEGG) pathways (Fig. 4).

Module “yellow” grouped together genes clearly upregulated only by LPS + IFNγ, such as the members of the GBP family: *Gbp1, Gbp8* and *Gbp10*. These IFNγ-inducible GTPases are involved in defence responses to viruses and bacteria^28^. Interestingly, our analysis of this module resulted in an enrichment of “defence response to virus and protozoan” together with “cytokine-mediated signalling pathway” and “cellular response to IFNγ” GO terms (Fig. 3A). Furthermore, “cytokine-cytokine receptor interaction”, “chemokine signalling pathway” and “JAK-STAT signalling pathway” are among the most enriched KEGG pathways (Fig. 4A). These results suggest that genes in “yellow” module are affected by IFNγ, rather than LPS.

Both treatments, LPS and LPS + IFNγ, markedly upregulated genes clustered in the “blue” module. Our analysis revealed that these genes are mostly involved in inflammatory and immune response, since 8 out of the 10 most enriched GO terms were related to these biological processes (Fig. 3B). Actually, the “blue” module grouped together prototypical pro-inflammatory genes such as *Tnf, Csf3, Il-6, Cybb, Ptgs2, Nos2, Il-1β, Il-12a* and *Il-12b*. In this module, not only does IFNγ greatly affect gene expression, but so does LPS, since “cellular response to interferon-gamma”, “LPS-mediated signalling pathway” and “Toll-like receptor signalling pathway” were among the top-10 annotated GO terms and KEGG pathways (Figs 3B and 4B).

Genes found in the “green” module, i.e., genes upregulated by LPS but not by LPS + IFNγ, were also involved in inflammatory and immune responses (Fig. 3C). Since LPS alone greatly induces gene expression in this module, “response to lipopolysaccharide” and “Toll-like receptor signalling pathway” stand out among the most enriched GO terms and KEGG pathways (Figs 3C and 4C). In contrast, genes downregulated only in response to LPS, and clustered in the “red” module, play a role in non-inflammatory processes like “transcription”, “metabolism” and “phosphorylation” (Fig. 3D). Our analysis showed that there were no significant enriched KEGG pathways in the “red” module.

The “black” and “turquoise” modules include genes whose expression is decreased by LPS + IFNγ or by both treatments, respectively. “Transcription”, “translation”, “transport”, “metabolism” and “phosphorylation” were the main biological processes affected in these modules (Fig. 3E,F). Furthermore, the “black” module KEGG pathways annotation revealed that genes with decreased expression after LPS + IFNγ treatment play important roles in phagocytosis (Fig. 4D). In fact, previous work by our group has demonstrated reduced microglial capacity to phagocytose bacteria after LPS + IFNγ treatment^11^, which is in accordance with this result. Meanwhile, analysis of genes downregulated by both LPS and LPS + IFNγ, clustered in the “turquoise” module, revealed “Huntington’s, Alzheimer’s and Parkinson’s disease” to be among the most significant affected KEGG pathways (Fig. 4E). Note that genes identified in this study and classified within these three pathways represent more than 60% of all the genes annotated in these diseases, indicating the high relevance of the data presented here.

### In microglia, LPS and LPS + IFNγ downregulate Huntington’s, Alzheimer’s and Parkinson’s disease-related genes

**Figure 5:**
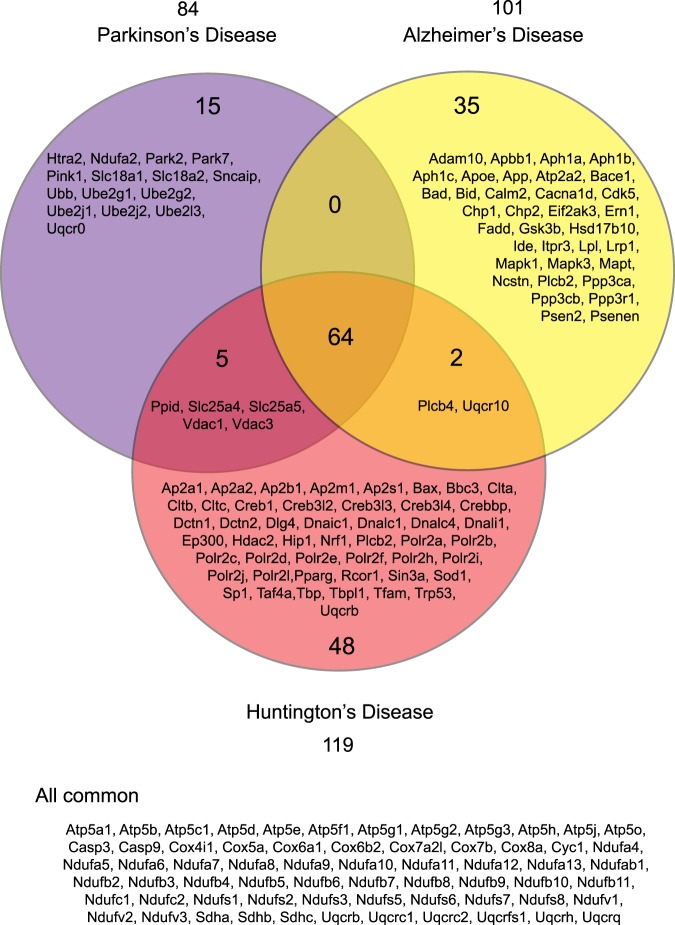
LPS and LPS + IFNγ downregulate 169 genes involved in Parkinson’s, Alzheimer’s and Huntington’s disease. The Venn diagram shows that as many as 64 of these genes are common to all three diseases. They belong to the gene families of ATP synthases, cytochrome c oxidases, NADH dehydrogenases, succinate dehydrogenases and ubiquinol-cytochrome c reductases. In turn, 15, 35 and 48 genes are specific to Parkinson’s, Alzheimer’s and Huntington’s disease respectively. Note the presence *Park2, Park7* and *Pink1* in Parkinson’s disease, *Adam10, Apoe, App, Bace1* and *Psen2* in Alzheimer’s disease and *Bax, Hdac2* and *Sp1* in Huntington’s disease.

Among the genes downregulated by the combination of both treatments, and sorted into the “turquoise” module, 119, 101 and 84 genes were annotated in Huntington’s, Alzheimer’s and Parkinson’s disease, respectively (Fig. 4E). In-depth analysis revealed that in total these represented 169 different genes. Of these, 64 were involved in all three neurodegenerative disorders, as depicted in the Venn Diagram (Fig. 5). Within these 64 common genes, there were different members of the gene families of ATP synthases, cytochrome c oxidases, NADH dehydrogenases, succinate dehydrogenases and ubiquinol-cytochrome c reductases. The Venn diagram also shows 15, 35 and 48 genes related exclusively to Parkinson’s, Alzheimer’s and Huntington’s disease, respectively.

*Park2, Park7* and *Pink1* stand out among Parkinson’s disease-specific genes downregulated by both treatments, since they are involved in biological processes characteristic of this disease, like dopamine uptake and neurotransmission, autophagy and degradation of mitochondria. Furthermore, our data reveal that *Adam10, Apoe, App, Bace1* and *Psen2* expression is decreased in microglial cells treated with LPS or LPS + IFNγ. These genes play a pivotal role in Nocht signalling, protein processing, apoptosis and beta-amyloid metabolic processes which are altered in Alzheimer’s disease. Finally, *Bax, Hdac2* and *Sp1*, involved in apoptosis and transcription, are found among the Huntington’s disease-specific genes affected by LPS and LPS + IFNγ.

### Differential expression of coding and non-coding RNAs induced by LPS and LPS + IFNγ

WGCNA revealed that although some genes respond equally to LPS and LPS + IFNγ, others do not. In order to further characterize microglial response to both treatments, we studied those RNAs that are differentially expressed using DESeq analysis. This is a straightforward approach to detect genes differentially upregulated or downregulated by direct pairwise comparisons.

Taking into account all the samples, 14,337 coding and non-coding RNA species were detected. When analysed separately, 11,225, 12,694 and 13,004 protein-coding RNAs were identified in Control, LPS and LPS + IFNγ samples, respectively (Table 2). Among non-coding RNAs, long non-coding RNAs (lncRNAs) were particularly abundant. Micro-RNAs (miRNAs) represented only 6% of the non-coding RNAs detected. Finally, pseudogenes, small nucleolar RNAs (snoRNAs) and small nuclear RNAs (snRNAs) were also found. Note that treatments can turn gene expression on or off; therefore, the number of RNAs detected is different for each Control, LPS and LPS + IFNγ condition.

**Table 2:** Number of protein coding and non-protein-coding RNAs detected in Control, LPS and LPS + IFNγ conditions.

	Control	LPS	LPS + IFNγ
Protein-coding	11225	12694	13004
Non-coding RNAs	1104	1174	1169
Pseudogenes	266	291	296
lncRNAs			
lincRNA	117	123	124
Antisense	140	144	140
Processed transcripts	126	128	124
Sense intronic	7	8	8
snoRNA	68	79	81
snRNA	7	7	9
miRNA	69	66	63
Miscellaneous RNA	52	53	52

In all three cases, protein-coding RNAs represent about 90% of RNAs detected. Note that because treatments can switch on or switch off gene expression, numbers are different when comparing conditions.

Data analysis revealed that LPS greatly influences protein-coding RNA expression, since 37% of these RNAs detected in primary microglia show significant changes compared to Control (Table 3). Upregulation versus downregulation is split roughly equally: 52.4% and 47.6% respectively. In addition, LPS turns on the expression of 32 protein-coding RNAs and turns off 27. LPS also tends to induce overexpression of non-coding RNAs, particularly snoRNAs and miRNAs, since none of them were downregulated by LPS.

**Table 3:** LPS and LPS + IFNγ markedly affect primary microglia gene expression.

	LPS vs Control						LPS + IFNγ vs Control						LPS + IFNγ vs LPS					
	Upregulated		Downregulated		TOTAL		Upregulated		Downregulated		TOTAL		Upregulated		Downregulated		TOTAL	
	n	ON	n	OFF	n	%	n	ON	n	OFF	n	%	n	ON	n	OFF	n	%
Protein-coding	**2456**	32	**2169**	27	4684	37%	**2312**	46	**2021**	18	4397	33.8%	**532**	1	**549**	3	1085	10%
Non-coding RNAs																		
Pseudogene	**13**	0	**4**	0	17	14%	**11**	0	**2**	0	13	10.8%	**1**	0	**2**	0	3	3%
Pseudogene polymorphic	**1**	0	**0**	0	1	33%	**1**	0	**0**	0	1	33.3%	**0**	0	**0**	0	0	0%
Pseudogene processed	**19**	0	**3**	1	23	16%	**17**	0	**2**	0	19	13.2%	**1**	0	**1**	0	2	2%
Pseudogene unprocessed	**7**	0	**5**	0	12	41%	**8**	0	**3**	0	11	37.9%	**1**	0	**1**	0	2	8%
lncRNA																		
lincRNA	**18**	1	**10**	2	31	25%	**18**	1	**9**	1	29	23.4%	**2**	0	**3**	0	5	4%
Antisense	**21**	1	**9**	0	31	22%	**14**	0	**6**	0	20	14.3%	**1**	0	**2**	0	3	2%
Processed																		
transcript	**14**	0	**6**	0	20	16%	**13**	0	**5**	0	18	14.5%	**2**	0	**2**	0	4	3%
Sense intronic	**1**	0	**0**	0	1	13%	**1**	0	**1**	0	2	25.0%	**0**	0	**0**	0	0	0%
miRNA	**5**	0	**0**	0	5	7%	**6**	0	**0**	0	6	9.4%	**0**	0	**0**	0	0	0%
snoRNA	**5**	0	**0**	0	5	6%	**0**	0	**0**	0	0	0.0%	**0**	0	**0**	0	0	0%
miscRNA	**27**	1	**13**	2	43	83%	**27**	1	**11**	2	41	80.4%	**0**	0	**5**	0	5	10%
Unknown	**16**	0	**7**	1	24	13%	**15**	0	**8**	0	23	12.2%	**3**	0	**3**	0	6	3%

DEGs in LPS versus Control, LPS + IFNγ versus Control and LPS + IFNγ versus LPS comparisons are showed. For each RNA species, the number of upregulated and downregulated RNAs is depicted. Switched ON/OFF RNAs are considered separately. Total genes affected are the sum of all four categories. Total percentage indicates the number of genes differentially expressed to the total number of genes detected in each RNA type.

Regarding expression changes induced by LPS + IFNγ when compared to Control, our results do not differ, in general terms, from those reported above for LPS: the percentage of affected RNAs and the numbers of those increased or decreased are similar, though snoRNAs are an exception. Comparison between LPS and LPS + IFNγ shows that 10% of coding RNAs are altered by IFNγ addition. Its effect can be synergistic or antagonistic to LPS, since the number of RNAs increased or decreased is similar.

**Figure 6:**
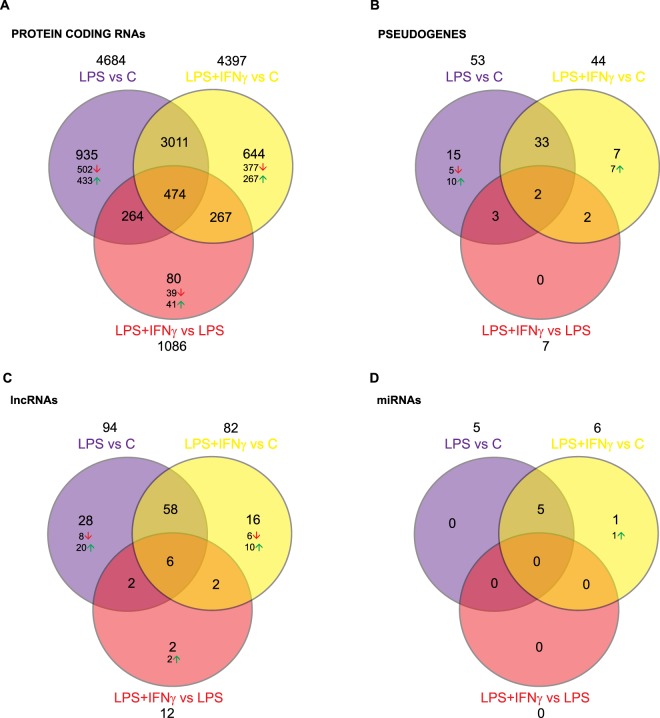
Differentially expressed RNAs affected by either or both of LPS and LPS + IFNγ. Venn diagrams showing the number of protein-coding RNAs (**A**), pseudogenes (**B**), lncRNAs (**C**) and miRNAs (**D**) significantly affected by LPS or LPS + IFNγ in primary microglia as assessed by DESeq analysis. Note the large number of protein coding genes significantly affected by both LPS and LPS + IFNγ; but also the existence of many genes specifically affected by LPS or LPS + IFNγ.

Venn diagrams (Fig. 6) also prove to be a useful tool to evaluate overall similarities and differences between the changes induced by LPS and by LPS + IFNγ in primary microglia. The response of protein-coding RNAs to LPS is stronger than that to LPS + IFNγ, since 4,684 and 4,397 genes are affected, respectively. However, most of them are common, specifically 3011. An additional 935 are affected by LPS alone, whereas only 644 are affected by LPS + IFNγ alone. In contrast, when comparing LPS + IFNγ directly with LPS (not each treatment with Control) our results show that out of 1,086 protein-coding RNAs altered. Of them, 80 are exclusively affected by LPS + IFNγ compared to LPS. Interestingly, there are 474 protein-coding RNAs whose expression changes in all three comparisons: LPS versus Control, LPS + IFNγ versus Control and LPS + IFNγ versus LPS.

The effect of LPS activation is also notable in pseudogenes and lncRNAs, where once again many LPS-altered RNAs also respond to LPS + IFNγ co-treatment. miRNAs are quite particular, since only 6 are differentially expressed: mir-155, mir-221, mir-222, mir-22hg, mir-5111 and mir-1957a. Five of these are induced by both LPS and LPS + IFNγ, but only mir-1957a expression increases due to LPS + IFNγ. As opposed to protein-coding RNAs, pseudogenes and lncRNAs, no miRNA changed when comparing the two treatments directly.

### LPS- and LPS + IFNγ-treated microglia share common features, but have distinct phenotypes

Little is known about the biological functions of non-coding RNAs in microglia, except for a few miRNAs, such as mir-155, which was upregulated by both treatments in our analysis. Therefore, we focused on protein-coding RNAs to determine the similarities and differences between LPS- and LPS + IFNγ-activated microglia.

**Figure 7:**
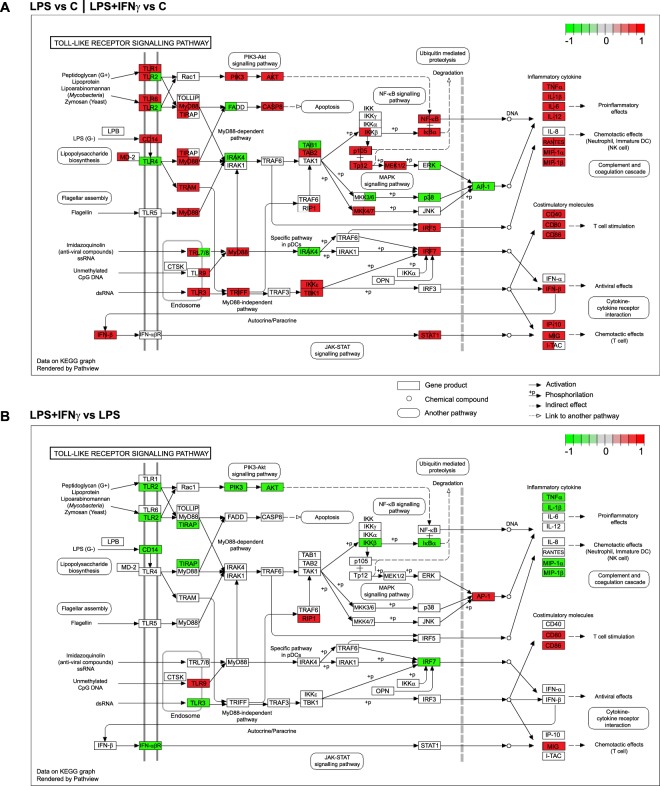
Toll-like receptor signalling pathway shows similarities, but also differences between LPS and LPS + IFNγ. (**A**) Genes involved in Toll-like receptor signalling affected by LPS (left side of the square) or LPS + IFNγ (right side of the square) compared to control. (**B**) This graph shows the genes within the pathway that are significantly altered when comparing LPS + IFNγ to LPS. Red indicates upregulation; green downregulation. Data on KEGG graph^23–25^ were rendered using Pathview.

SPIA analysis of the DEGs^26^ provides the first clues to this question. We analysed separately all 4,684, 4,397 and 1,086 DEGs affected in the LPS vs Control, LPS + IFNγ vs Control and LPS + IFNγ vs LPS comparisons, respectively. The Toll-like receptor signalling pathway was significantly affected in all of them. Interestingly, many genes involved in this pathway were upregulated by both LPS and LPS + IFNγ treatments (Fig. 7A). However, others including *Tlr2, Tlr7* and *Tlr8* were downregulated by LPS + IFNγ. A dampening effect of IFNγ was also observed in the case of inflammatory genes such as *Tnf*, *Il-1β*, *IKKβ* and *Irf7*, when comparing LPS + IFNγ to LPS (Fig. 7B).

**Figure 8:**
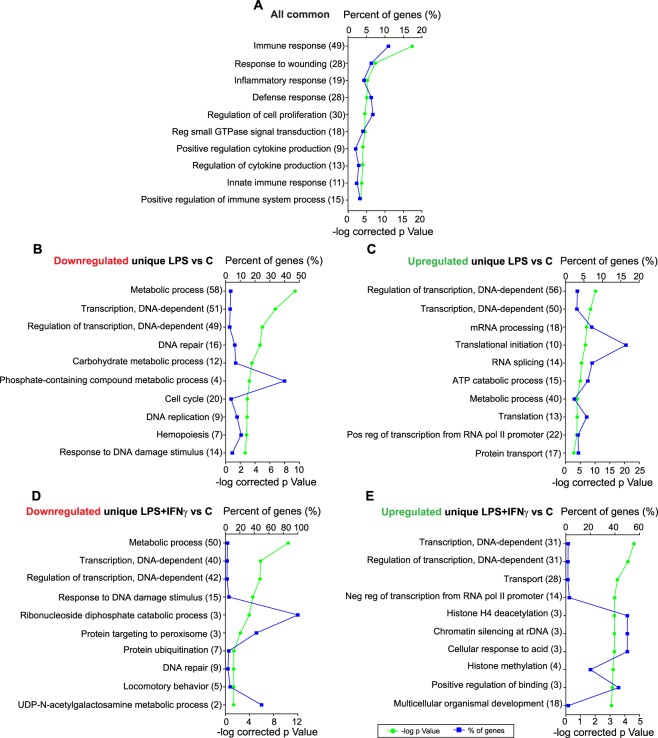
LPS and LPS + IFNγ are involved in similar biological functions, but some differences exist. This figure shows the 10 most enriched GO terms in: (**A**) genes common to LPS vs Control, LPS + IFNγ vs Control and LPS + IFNγ vs LPS (n = 474); (**B**) Genes only downregulated in LPS vs Control (n = 502). (**C**) Genes only upregulated in LPS vs Control (n = 433). (**D**) Genes only downregulated in LPS + IFNγ vs Control (n = 377). (**E**) Genes only upregulated in LPS + IFNγ vs Control (n = 267). Note that 7 of the 10 GO terms in (**A**) are related to immune and inflammatory responses. (**(B–E)** Share metabolism and transcription as the biological processes affected; but LPS tends to alter cell cycle (**B**) and LPS + IFNγ chromatin modifications (**E**). Gene percentage annotation (i.e., the percentage of the total number of genes annotated in each category detected in this work) is depicted on the upper x-axis, whereas the p-value is represented on the lower x-axis. The GO terms and the number of genes annotated within each of them are presented on the y-axis.

More significant results were obtained when considering common and non-common DEGs separately. *Nos2, Tnf, Tlr2, Tlr3, Il-1β, Il-18, Il-23a, Irf1, Irf7* and *Irf8* were among the 474 coding RNAs differentially expressed in all three comparisons. These RNAs did not share the same expression profile, e.g., *Nos2* expression increases due to both treatments but particularly with LPS + IFNγ; while LPS alone induced the highest *Tnf* expression. These findings are in accordance with the qRT-PCR results (shown in Fig. 1A). This expression heterogeneity is also confirmed by WGCNA: 30%, 26%, 20%, 16%, 5% and 3% of the DEGs detected by DESeq are clustered within the “yellow”, “green”, “blue”, “turquoise”, “pink” and “red” modules, respectively. Even so, most of them are involved in inflammatory and immune responses, since 7 of the 10 most significant annotated GO terms were related to these processes (Fig. 8A). Thus, both treatments, LPS and LPS + IFNγ, trigger an inflammatory microglial phenotype.

Regarding non-common DEGs, 935 RNAs were affected only by LPS alone and 644 by LPS + IFNγ compared to Control. Both treatments seem to be more prone to inhibit expression of these RNAs than to promote it. Thus, 502 RNAs were downregulated and 433 were upregulated due to LPS, whereas expression of 377 RNAs was decreased and that of 267 was increased in response to LPS + IFNγ (Fig. 6A). Once again, these expression patterns were in accordance with WGCNA results. 97% and 3% of genes downregulated only by LPS were found within the “turquoise” and “red” modules, whilst most of the upregulated genes were clustered in the “green” and “blue” modules. Meanwhile, the “turquoise” and “black” modules included all the genes whose expression decreased only as a result of LPS + IFNγ treatment; whereas the genes upregulated only by this treatment were clustered in the “blue”, “yellow” and “pink” modules. According to the GO analysis, most of the DEGs only affected by LPS or only by LPS + IFNγ were involved in transcription and regulation of transcription processes (Fig. 8B-E). Furthermore, since inflammatory and immune responses are also associated with shifts in metabolism^29^, metabolic processes were also greatly altered by LPS and LPS + IFNγ.

**Figure 9:**
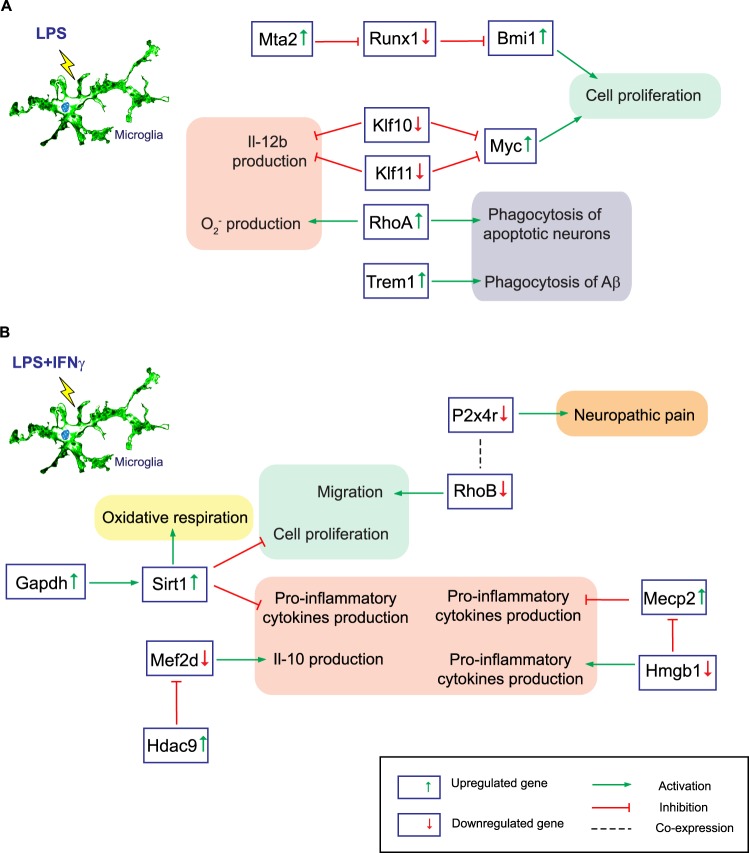
A model of specific signalling pathways triggered by LPS and LPS + IFNγ in primary microglia. (**A**). By upregulating genes like *Mta2, Bmi1, Myc, RhoA* and *Trem1* and downregulating *Runx1, Klf10* and *Klf11*, LPS promotes microglial proliferation, phagocytosis and the release of pro-inflammatory cytokines. (**B**). LPS + IFNγ induces expression of *Gapdh, Sirt1, Hdac9* and *Mecp2*, whereas it reduces that of *Mef2d, P2xr4, RhoB* and *Hmgb1*. This suggests that LPS + IFNγ-treated microglia have less proliferative potential, show reduced expression of genes involved in neuropathic pain and are more prone to resolve inflammation by activation of oxidative respiration and blockade of pro-inflammatory cytokine production.

Although these results suggest that LPS and LPS + IFNγ-treated primary microglial cultures are similar, note that the genes involved in the biological processes are different for each condition. On one hand, we found that LPS alone significantly downregulates the transcription factors *Runx1, Klf10* and *Klf11*, but upregulates the small GTPase *RhoA*, oncogenes *Myc* and *Bmi1*, chromatin remodelling factor *Mta2* and membrane receptor *Trem1* (Fig. 9A). On the other hand, LPS + IFNγ significantly decreases expression of the small GTPase *RhoB*, membrane receptor *P2x4r*, transcription factor *Mef2d* and non-histone nuclear DNA-binding protein *Hmgb1*. Additionally, LPS + IFNγ also increases the expression of the moonlighting protein *Gapdh*, sirtuin *Sirt1*, histone deacetylase *Hdac9* and epigenetic regulator *Mecp2* (Fig. 9B).

Finally, only 80 RNAs were differentially expressed when directly comparing LPS + IFNγ to LPS. Of these, 39 were downregulated and clustered in the “black” (82%) and “turquoise” (18%) modules. The remaining 41 were upregulated and found within the “red” (94%) and “pink” (6%) modules (Fig. 6A). Because of the small number of affected genes, little can be inferred from analysis of the GO terms associated with these genes (data not shown).

## Discussion

LPS alone or together with IFNγ is widely used to trigger microglial activation. However, to the best of our knowledge, similarities and differences between the effects of these two treatments on microglial gene expression have not been fully characterized. In the present study, to address this, we analysed the transcriptomic profile of primary microglial cells in response to LPS and LPS + IFNγ by RNA-Seq.

Determination of the time point used in this study was a significant initial step. Evidence shows that not all genes are expressed at once, but each has its own specific temporal expression pattern. Using LPS-treated primary microglia and BV2 cells, Das and colleagues^12,30^ reported that the most significant expression changes occur within the first and the eighth hour after treatment. We also previously described a marked gene upregulation in cultured microglia treated with LPS and LPS + IFNγ for 6 h^11,31,32^. In this study, our qRT-PCR results showed significant alterations of canonical pro-inflammatory genes, such as *Tnf, Csf3, Il-6, Cybb, Ptgs2* and *Nos2*, 6 hours after treatment. The expression profile of these genes was also observed after analysing RNA-Seq data, thus validating the high-throughput sequencing.

We are aware that analysing a single time point is a limitation of this study. Nevertheless, the data presented here for 6 hours after treatment offer a good picture of the first wave of transcriptional changes induced by LPS or LPS + IFNγ which are mainly regulated by latent transcription factors^33^. The relative timing of IFNγ and LPS exposure was also important. We decided to use co-treatment because this procedure results in a strong potentiation of LPS-induced NO production and neurotoxic effects^10,34^. Priming with IFNγ could have resulted in a somewhat different pattern of modulation, including a stronger potentiation on the production of some pro-inflammatory cytokines like TNFα^35^.

Genes or proteins that are directly linked might have a similar function and therefore may be part of the same biological pathway. More and more, network methods such as WGCNA are chosen to analyse these interactions^20^. This approach classifies the whole transcriptome into discernible expression patterns. Furthermore, it compares all genes in the data and takes into account all changes through all samples at the same time. Therefore, it can extract complex expression profiles that could be easily missed if only DESeq pairwise differential expression analysis was used to examine the biological relevance of RNA-Seq data. Therefore, we used WGCNA to organize gene expression data in order to establish clusters or modules of genes with similar responses to LPS and LPS + IFNγ treatments. Our results show that these two stimuli, both considered pro-inflammatory, do not affect all transcripts in the same way, since 11 different gene expression patterns were detected. The results also show that while some genes are affected by both treatments, others are only affected by LPS or LPS + IFNγ, which means that LPS and LPS + IFNγ do not always affect a given gene in the same way. We consider these observations to be relevant findings. Frequently, reports only focus on gene upregulation due to pro-inflammatory stimuli^12,36^. However, according to our data, 9,343 genes out of 14,337 tend to be downregulated, without this trend emerging as statistically significantresult, by both treatments. Furthermore, in other studies, microglia are only treated with LPS + IFNγ, as strong pro-inflammatory stimuli, without considering a potential antagonistic effect between LPS and IFNγ^37,38^.

According to GO analysis, the genes that are upregulated by pro-inflammatory stimuli in cultured microglia show a significant enrichment of GO terms related to inflammatory and immune responses. The annotation of these functions in response to LPS and/or LPS + IFNγ is interesting and indicates that our approach of comparing both treatments is strong but rather predictable. To the best of our knowledge, a comprehensive transcriptional profile of LPS + IFNγ-treated microglia has not been derived using RNA-seq, though the mouse embryonic stem cell-derived microglia response to this stimulus has been analysed via RNA microarrays^39^. Those data showed an exacerbated immune response and increased pro-inflammatory gene expression. Other reports also identify alterations in inflammatory and immune system processes, as well as a marked upregulation of pro-inflammatory genes, such as *Ptgs2, Tnf, Il-12a and Il-1β*, in the BV2 microglial cell line, primary microglial cultures and acutely isolated microglia from adult CNS treated with LPS (Table 4). Altogether, this is in agreement with our results.

**Table 4:** Relevant previous microglial transcriptomic studies.

Reference	Microglia source	Treatment conditions	Main findings
^12^	BV2 microglial cell line	LPS for 2 and 4 h	367 DEGs, 2 h after LPS treatment, 263 ↑ and 104 ↓. 512 DEGs, 4 h after LPS treatment, 319 ↑ and 193 ↓. Most induced genes, such as *Nos2, Il-1α, Il-1β, Tnf, Ptgs2, Ir1, Ir7, Ir9* and *Cxcl10*, were involved in immune system, response to stimulus, multiorganism processes and death biological functions.Markedly decreased genes, like *Irak1, Cxcr4, Hdac9* and *Tlr8*, participate in regulation of biological and cellular processes and development regulation processes.
^30^	BV2 microglial cell line Mouse primary microglia (PM)	LPS for 2 and 4 h	BV2 microglial cell line is poorly representative of PM. In response to LPS a much larger number of transcripts was altered in PM than in BV2: In PM, 531 DEGs were affected 2 h after LPS treatment (362 ↑, 169 ↓), whereas 4 h treatment altered 1286 DEGs(946 ↑, 340 ↓). Exposure to LPS altered 237 DEGs (205 ↑, 32 ↓) after 2 h and 331 DEGs (299 ↑, 32 ↓) after 4 h, in BV2. Still, some similarities were observed, e.g. BV2 and PM shared 142 and 264 upregulated DEGs at 2 and 4 h, respectively. Furthermore, the top 150 upregulated genes after 4 h LPS exposure were involved in immune system, multiorganism processes, locomotion, response to stimulus and cell killing processes in both cell types.
^39^	Mouse primary microglia Mouse embryonic stem cell-derived microglia (ESdM)	LPS + IFNγ for 16 h IL-4 and TGF-β for 48 h LPS + IFNγ, IL-4 and TGF-β for 24 h	In ESdM, the strongest transcriptome changes were induced by LPS + IFNγ. Most upregulated genes include *Nos2, Tnf, Socs1, Socs3, Stat1, Marco, CD80* and *Icam1*, whereas *Arg1* and *Mrc1* were downregulated. LPS + IFNγ GO-terms were related to immune functions. IL-4 GO-terms related to metabolic and catabolic processes. TGF-β GO-terms linked to angiogenesis, cell motility and metabolism. ESdM closely resembles PM on whole transcriptome, although response to LPS + IFNγ was weaker.
^72^	Ovine foetus primary microglia	LPS for 6 h	258 DEGs due to LPS treatment were found. Of them, 205 were upregulated (e.g. *Il-1a, Irf7, Jak3, Ptges*) and 53 were downregulated (*e.g. C/EBPα, Hmox1, Cxcr4*) LPS upregulated NFκB, PIK3-Akt and JAK-STAT inflammatory pathways, but downregulated metabolic processes
^73^	Human primary microglia	IFNγ for 1 h + LPS for 48 h (M1) IL-4 + IL-13 for 48 h (M2a) IL-10 added to M2a at 24 h + 144 h (M2c) TGF-β added at 24 h + 120 h (Mtgf)	Mtgf closely resembles basal microglia (M0). M1 transcriptome most distinct from M0 (837 genes were induced, including mir-155, and 618 were reduced). M1 stimuli lead to an almost complete loss of myelin phagocytic activity (Mtgf > M2c > M2a = M0 > M1)
^54^	Acute isolated cortical microglia from adult mouse	Intraperitoneal LPS for 24 or 48 h	10788 genes were detected. Of them, 4179 were significantly altered by LPS (50% ↑, 50% ↓). Top-3 deregulated pathways were Eif2 signalling, mitochondrial dysfunction and protein ubiquitination pathway. Affected inflammatory pathways include Il-10, Tnfr2 and NFκB signalling pathways. LPS also altered non-inflammatory pathways, such as androgen signalling and Huntington’s disease. Cell death and survival, cellular growth and proliferation, cellular function and maintenance and gene expression were among most enriched GO-terms. However, 38% of deregulated genes were related to the microglia ability to sense their environment.
^74^	Acute isolated microglia from adult mouse CNS	Intraperitoneal LPS for 4 h Aging models: Physiological aging (4 and 24 months) Ercc1, DNA damaged induced aging Disease models: AD, App-Ps1 Amyotrophic Lateral Sclerosis, Sod1	Aging and neurodegeneration share a gene expression profile, particularly in upregulation, which substantially differed from the inflammatory gene network induced by LPS. Genes altered in aging and neurodegeneration were involved in immune, phagosome, lysosome, oxidative phosphorylation and antigen presentation signalling pathways. LPS enriched for ribosome, TLRs and NOD-like receptor signalling pathways. Despite their dissimilarities, *Tlr2*, *Il-1β*, *Cxcl10* and *Spp1* genes, related to innate immune response, were upregulated in all 5 models.

The effect of LPS and LPS + IFNγ on microglia transcriptome has been previously addressed using different cell sources: BV2 cell line, primary culture, mouse embryonic stem cell-derived microglia or acute isolated microglia from adult CNS. Reports describe an exacerbated inflammatory and immune response due to the treatments, as well as upregulation of pro-inflammatory genes. Furthermore, genes involved in metabolic processes tend to be downregulated by LPS. These findings are in accordance with the results here presented. Comparison between LPS and LPS + IFNγ-treated microglia transcriptomes was not the central topic of these studies.

Downregulated genes due to pro-inflammatory treatments are often overlooked because they are not thought to be involved in inflammation^12,36^. Here, we show that most genes expressed in non-activated primary microglia are repressed when these cells are activated by LPS or LPS + IFNγ. Transcription, translation, metabolism, transport, apoptosis and phosphorylation were the main biological processes in which the genes that are downregulated by one or both treatments were involved. These results relativize the concept of resting microglia in physiological conditions.

Interestingly, 169 genes downregulated by both treatments were involved in three neurodegenerative pathologies: Huntington’s, Alzheimer’s and Parkinson’s disease. Common features of these disorders include abnormal protein aggregation, oxidative stress and mitochondrial dysfunction^40^. Indeed, 64 downregulated genes play a role in all three diseases; most of them belong to gene families involved in ATP synthesis through the mitochondrial electron transport chain. According to previous reports, inhibition of the mitochondrial respiratory chain enhances mitochondrial ROS production^41^. Therefore, downregulation of genes involved in the electron transport chain due to LPS and LPS + IFNγ treatments in microglia could lead to cellular damage and death of neighbouring cells due to ROS overproduction. Thus, activating primary microglia with both stimuli could be an experimental model to study this process.

CNS inflammation plays a role in the progression of chronic neurodegenerative diseases and this inflammatory response is mostly governed by microglia^42^. In agreement with our results, LPS downregulates the E3 ubiquitin ligase *Park2* in microglial cells^43^, which leads to the accumulation of Tnf, Il-1β and Nos2. *Park7*-deficient microglia also show higher levels of Tnf, Nos2 and Ptgs2^44^. Finally, *Pink1* deletion exacerbates microglial activation^45^. Thus, downregulation of these genes could promote a pro-inflammatory microglial phenotype, thereby contributing to neurodegeneration in Parkinson’s disease.

Activated microglia induce neuronal cell death through the release of cytotoxic factors including cytokines and ROS in Alzheimer’s disease^46^. Interestingly, the “cytokine activity”, “defence response” and “T-cell activation” GO terms were associated with upregulated genes in an RNA-Seq analysis performed in sorted microglia from 15 to 18 month-old APP/PS1 transgenic mice^47^. Furthermore, that report showed downregulation of *ApoE* expression, similar to our data presented here. It was possible to revert this process, which is highly impaired in Alzheimer’s disease, by co-culturing neonatal microglia in brain slices of 20 month-old APP/PS1 mice. Therefore, a comparison between young and old microglia transcriptomic profiles could provide some clues as to which processes are impaired with age and lead to disease. Little is known about *Adam10, App, Bace1* and *Psen2* microglial expression. Nevertheless, due to their involvement in Alzheimer’s disease pathophysiology, it would be worth further studying the implications of their downregulation in response to LPS and LPS + IFNγ. Similarly, decreased expression of the Huntington’s disease-related genes *Sp1, Hdac2* and *Bax*, as a result of both treatments could provide some clues about the underlying disease mechanisms.

This study was performed with primary microglial cultures isolated from neonatal mouse cortex. Although this model is widely used and has contributed considerably to our current knowledge on microglial biology, it is important to be aware of its limitations. As with most cell types, *in vitro* culture has an impact on microglial biology. Gosselin and colleagues^48^ reported significant transcriptomic changes in human and mouse brain microglia when cultured. The transfer of cells to the *in vitro* environment resulted in 10-fold induction of more than 300 genes and 10-fold reduction of more than 700 genes after 7 days. Although cultured microglial cells show an activated phenotype, they become less responsive after 13–16 days *in vitro* (DIV)^49,50^. The microglial cultures in this study were used at 21 DIV. This relatively long time in culture allows microglia to mature and become less reactive, particularly because of their interactions with astrocytes. Be that as it may, it is important to acknowledge that the lack of neuronal inhibitory inputs probably results in microglia being in a more activated state than in the adult CNS *in vivo*. Another caveat to be added to this study is that microglial cells were isolated from the brains of early neonates. This allows for the preparation of microglial cultures with a high yield in a simple and reproducible manner. However, since early postnatal microglial cells differ from adult or aged microglia in terms of morphology, function and gene expression^51^, caution must be exercised when interpreting our data. Recent improvements in the isolation of fresh microglia using fluorescence-activated cell sorting and laser capture microdissection methods have been reported^52^. Such methods have the advantage over cultures that microglial cells live in their physiological environment until the moment of extraction, but they are not free from artefacts. The isolation procedure for fluorescence-activated cell sorting requires 3 to 5 hours, which may affect the expression profile of microglia. In contrast, cellular lysis in cultured microglia is immediate and the transcriptome obtained faithfully reflects the transcriptome of the living cells, which also occurs with RNA from microglia isolated by laser capture microdissection. A main drawback of RNA from microglia isolated by laser capture microdissection is the probable presence of RNA from surrounding cells. In this respect, the purity of cultured microglia RNAs is far superior. As has been stated before, it is critical to be aware of the limitations of the protocol used and choose the method most appropriate for the scientific question addressed^53^. Despite their limitations, LPS- or LPS + IFNγ-treated microglial cultures allow a considerable experimental versatility and provide valuable information on the potential phenotypes of activated microglia.

Apart from revealing the inhibition of important genes implicated in neurodegenerative diseases in cultured microglial cells treated with LPS or LPS + IFNγ, the present RNA-Seq analysis also allowed us to gain further understanding of how both stimuli affect microglial gene expression. Thus, DEGs were obtained using DESeq analysis. Both protein-coding and non-protein-coding RNAs were found; although protein-coding transcripts represented 90% of all RNAs detected. Among the non-protein-coding RNAs, few miRNAs were detected because the sequencing protocol used here only allows for the detection of pre-miRNAs. Thus, the data presented here show only a small fraction of the miRNAs present in primary microglial cells. To conduct a specific study of miRNAs, another sequencing library preparation protocol should be used.

LPS and LPS + IFNγ have a slightly greater tendency to upregulate gene expression than to downregulate it in all the transcripts differentially expressed. This slight difference between the number of genes upregulated and those downregulated, though in accordance with Hirbec *et al*.^54^, differs from most previous microglial RNA-Seq analysis, where most of the affected genes were upregulated (Table 4). These differences may be due to different time points, treatment doses or microglia sources being used. The number of samples can also lead to discrepancies^30^.

Because information on non-protein-coding RNAs is scant, the functional significance of their expression changes due to treatments in microglial biology was not further considered in this work. Instead, we focused on protein-coding RNAs. LPS versus Control and LPS + IFNγ versus Control comparisons shared more than 3000 DEGs. Moreover, another 474 DEGs were common to these two comparisons and also to the LPS + IFNγ versus LPS comparison. Most of these genes, including *Nos2, Tnf, Tlr2, Tlr3, Il-1β, Il-18, Il-23a, Irf1, Irf7* and *Irf8*, are involved in inflammatory and immune responses, thus validating the usefulness of LPS and LPS + IFNγ to induce and study microglial activation *in vitro*. For this reason, they are sometimes used interchangeably. A detailed look into the genes differentially affected only by LPS and LPS + IFNγ, together with a comprehensive review of the literature, has allowed us to better understand the different signalling pathways affected under each condition.

The transcription factor *Runx1* is sufficient to inhibit microglia activation in response to LPS treatment. *Runx1* acts in amoeboid microglia to limit the length of the activated phase and guide the cells toward a deactivated phenotype. Moreover, this transcription factor inhibits the proliferative capacity of amoeboid microglia^55^. Through its target *Bmi1, Runx1* is involved in cell self-renewal and its depletion increases *Bmi1*, thereby inducing cell proliferation^56^. It seems probable that, LPS downregulates *Runx1* expression by increasing *Mta2*: a member of NuRD complex^57^. Therefore, *Bmi1* expression is upregulated, which suggests the existence of a proliferative microglial phenotype in response to this treatment (Fig. 9A). The activation of the oncogene *Myc* also supports this hypothesis. *Klf10* and *Klf11* decrease cell proliferation and induce apoptosis by targeting *Myc*^58^. LPS treatment not only downregulates *Klf10* and *Klf11* genes in microglia, but also in bone marrow-derived macrophages. This inhibition enhances *Il-12b* expression^59^. Inflammation is also elicited by the upregulation of the small GTPase *RhoA*, since it promotes superoxide release^60^. *RhoA* also induces microglial phagocytosis of apoptotic neurons^61^, whereas *Trem1* favours microglial Aβ uptake^62^. Therefore, LPS triggers a proliferative, pro-inflammatory and phagocytic microglial phenotype.

The NAD-dependent deacetylase *Sirt1* is a crucial regulator of energy metabolism and cell survival. It supports oxidative respiration and anti-inflammatory responses, thus enhancing the resolution of inflammation^63^. *Gapdh* promotes *Sirt1* activation and autophagy initiation which protect cells from apoptosis induced by glucose withdrawal^64^. Thus, through *Gapdh*, LPS + IFNγ could enhance *Sirt1* expression (Fig. 9B). LPS + IFNγ-activated microglia seem to be non-proliferative and non-motile, since *Sirt1* blocks microglial proliferation^65^ and the small GTPase *RhoB*, which induces cell migration, is downregulated by the co-treatment^66^. After spinal cord injury, *RhoB* is co-expressed with the purinergic receptor *P2x4r* in microglial cells. This receptor is a major contributor to the development of neuropathic pain. Therefore, *P2x4r* downregulation due to LPS + IFNγ could be related to pain reduction. This stimulus is also involved in chromatin structure. *Sirt1* and *Hdac9* are histone deacetylases and *Mecp2* participates in histone methylation. All three upregulated genes participate in inflammatory responses. *Sirt1* suppresses the release of pro-inflammatory cytokines^65^. Meanwhile, *Hdac9* binds and represses the transcription factor *Mef2*^67^, which in later stages of microglial activation promotes IL-10 release^68^. Finally, *Hmgb1* interacts with and represses *Mecp2*^69^. However, LPS + IFNγ downregulates *Hmgb1*, thus increasing *Mecp2* expression and blocking secretion of pro-inflammatory mediators^70,71^. Thus, these results suggest that LPS + IFNγ treatment reduces genes involved in neuropathic pain, as well as microglial migration and proliferation, whereas it also promotes some anti-inflammatory responses.

Altogether, it seems that the addition of IFNγ could, somehow, dampen some pro-inflammatory genes. Downregulation of inflammatory mediators and effectors, such as that of Tnf, Il-1β, IKKβ and Irf7 observed in Toll-like receptor signalling pathway analysis when comparing LPS + IFNγ and LPS, supports this hypothesis. To confirm it, an additional experimental group, IFNγ-treated microglia, should have been included. This is certainly a limitation of the study.

## Conclusions

In summary, by using RNA-seq, we have compared the transcriptomic changes induced by LPS and LPS + IFNγ in primary murine microglia. Our study demonstrates that both treatments greatly affect microglial gene expression. However, not all genes are equally affected by LPS and LPS + IFNγ. Interestingly, both treatments downregulate genes involved in Parkinson’s, Alzheimer’s and Huntington’s disease. These data break new ground in the study of the role of microglia in these disorders. Furthermore, a detailed look at the genes differentially expressed only by LPS or LPS + IFNγ has revealed that LPS-treated microglia are proliferative, phagocytic and release pro-inflammatory cytokines. In contrast, LPS + IFNγ reduces cell proliferation, downregulates genes involved in pain and favours resolution of inflammation. These different profiles must be taken into account when using LPS or LPS + IFNγ in *in vitro* models of microglial activation.

### Ethics approval

All procedures involving animals were approved by the Ethical Committee for Animal Experimentation of the Universitat de Barcelona and by the Commission for Animal Experimentation of the Generalitat de Catalunya, with protocols numbers DAAM 5026, 7064 and 7065.

## Data Availability

RNAseq datasets were deposited in the Gene Expression Omnibus database under dataset accession number GSE90046. Note that this dataset also includes samples from our previous work^11^. However, no overlap analysis exists between both reports. Samples here analyzed can be found as C/EBPβ^fl/fl^ genotype, considered wild-type. Other data supporting the conclusions of this study will be available upon reasonable request.
